# Artificial immune systems (GA-AIS) enabled power loss mitigation in distributed generation: X3PAIS optimization approaches

**DOI:** 10.1016/j.heliyon.2024.e37332

**Published:** 2024-09-06

**Authors:** Oday A. Ahmed, K.H. Chong, S.P. Koh, Chong Tak Yaw, Jagadeesh Pasupuleti

**Affiliations:** a Department of Electrical Engineering, University of Technology- Iraq, 35299, Baghdad, Iraq; b Institute of Sustainable Energy, Universiti Tenaga Nasional (The Energy University), Jalan Ikram-Uniten, Kajang, 43000, Selangor, Malaysia

**Keywords:** Artificial immune system, Distribution generation, Genetic algorithm, Power loss reduction

## Abstract

The distribution network is a crucial component of the power system, with industrialization driving increased energy demand. Traditional power-generating techniques, such as thermal and hydroelectric are not enough to meet this demand, leading to the development of Distributed Generation (DG). DG requires an extensive re-evaluation of the current power system, as it modifies energy losses and line flows. Inadequate integration of DG can cause power outages, disruption of protection coordination, and lead to islanding. AI can help overcome this issue by determining the best system architecture. Researchers have been interested in the Artificial Immune System (AIS) algorithm, which has room for development and lacks a fixed template. In order to improve AIS, X3PAIS, a hybridization strategy that combines clonal selection with a three-parent crossover has been developed within the scope of the study. X3PAIS was pre-tested using applications in a planetary gear train, a wastewater treatment facility, and mathematical calculations, showcasing its robustness and versatility. In the context of power distribution, X3PAIS is used in the multiple DG architecture of the power distribution system, reducing power losses by placing DG units in the best locations and sizing them to match load profiles. The four DGs' experiment results show that X3PAIS can minimize power losses by more than 89 %. To optimize power losses in the power distribution system, X3PAIS may be improved with a three-parent multiple-point crossover operation.

## 1 Introduction

**Fig. 1:**
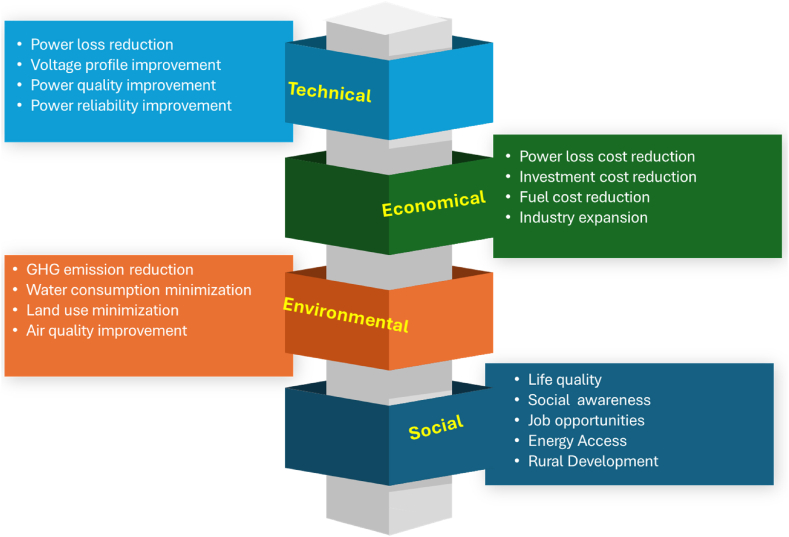
Numerical advantages of DG adoption [4].

The use of Distributed Generation (DG) enhances electricity quality and dependability, while simultaneously decreasing production costs and carbon emissions [1]. In addition, the use of DGs in power plants has been expedited by advancements in compact generators, Uninterruptible Power Supply (UPS), storage devices, and power electronic devices [2]. DG units provide cost benefits in terms of fuel investment, resulting in reduced power costs and improved voltage stability and margin within the system [3]. These generating technologies may also be used by utility companies to enhance the diversity of their energy portfolio and transform electric power networks into self-governing and intelligent systems. The incorporation of DGs into the Distributed Network (DN) has seen significant growth due to several advantages in terms of technology, economy, environment, and society, as shown in Fig. 1. Wind turbines, Photovoltaic (PV) systems, fuel cells, small hydropower facilities, and reciprocating engines are examples of DG sources. The capacity of the DG ranges from a few kilowatts to many tens of megawatts. DG sources are not centrally scheduled or dispatched inside a Distributed System (DS). DG technologies are classified according to the specific power they are capable of supplying to the system. The DG technologies have been classified into four distinct categories (see Table 9).

(a) Type 1: The device is capable of just supplying active power to the system. PV, fuel cells, and microturbines are among the several types of DG systems.
(b) Type 2: It is capable of providing both active and reactive power to the system at the same time. Type 2 DG includes Double Fed Induction Generators (DFIGs) used in wind farms, as well as their synchronous counterparts.
(c) Type 3: The system is limited to receiving only reactive electricity. One instance of a type 3 DG is synchronous compensators, namely gas turbines.
(d) Type 4: It can provide active power; it also drains reactive power from the system. Induction generators used in wind farms are a common example of a type 4 distributed generator.

Researchers are motivated to further examine potential solutions due to the notable power loss and weak voltage profile issues seen in radial distribution systems. Furthermore, it is essential to address two other crucial concerns, namely the proper location and size of DG, before its integration into the grid system. The improper size and placement of DG might impede the regular functioning of the system. The integration of renewable-based DG remains the optimal approach for enhancing bus voltage and mitigating system losses [5]. Several analytical approaches are suggested in Refs. [6,7] to find the best size and placement of the necessary DG units in order to accomplish this goal. Along with those strategies, many AI and hybrid approaches are also covered in Ref. [8]. The Differential Evolution Algorithm (DEA) was developed by Sulkuti et al. [9] as a solution for power loss reduction concerns in a distribution system that incorporates network reconfiguration, DG, and distributed STATCOM. The simulation is conducted using the IEEE-69 bus system, and the generated findings demonstrate an 82.92 % decrease in power losses. In addition, the voltage profile of the bus with the lowest strength is enhanced from 0.9805 per unit to 0.9085 per unit.

As already discussed, the use of DG inside a power distribution system has the potential to impact several aspects such as short circuits, output voltage, line losses, system stability, and dependability. Studies have shown that improper positioning of DG may result in line losses that surpass those without DG. Direct current may mitigate line losses, but scaling can amplify losses. DG has the potential to enhance voltage levels when positioned at a low-voltage bus, but it may exceed the allowable voltage limitations when situated at a high-voltage node. The use of the Clonal Selection based Artificial Immune System (AIS) aims to mitigate power loss via the implementation of DGs inside the system [[10], [11], [12], [13], [14], [15]].

In Distributed Generation (DG) systems, the investigation and incorporation of hybrid optimization techniques, especially those that combine Artificial Immune Systems (AIS) and Genetic Algorithms (GA) were hardly studied. Individually AIS and GA have both been extensively studied and implemented in several applications, however, their combined potential has yet to be a research matter to be explored and fully realized its' potential [[16], [17], [18]]. Since DG networks are complex and dynamic systems with a wide range of operational variables and interdependencies, traditional optimization techniques frequently cannot handle their complexity. While single-method optimization techniques are the main focus of most studies, they are not as flexible and robust as those required to effectively decrease power losses and improve the placement and operation of DG units. Furthermore, security research demonstrates the applicability and effectiveness of advanced hybrid algorithms, such as the proposed GA-AIS, in diverse and practical scenarios. In this work, the X3PAIS algorithm, which combines the strong search methods of GA with the adaptive learning capabilities of AIS, offered an improved brand-new three-parent crossover operation system, has been implemented to overcome the issues related to the individual GA, and AIS. This research confirms the algorithm's versatility and durability by validating it across a range of applications, including planetary gear trains and wastewater treatment facilities which helps to optimize DG systems in a way that has not been possible with previous methodologies. The X3PAIS algorithm is an innovative optimization approach that enriches the traditional Artificial Immune System (AIS) by incorporating genetic algorithm principles, particularly through a three-parent crossover method [19]. The initialization step of the method generates an initial population of candidate solutions, each encoded as antibody representations similar to those found in the body's immune system. Next, each potential solution's performance is assessed using an objective function (e.g., power loss minimization in distributed generation systems) that gauges its fitness. Each candidate's affinity is determined by this fitness value, where a higher affinity denotes a better solution. Cloning is a procedure that replicates the natural immune response by selecting candidates with better affinity to produce many copies of strong candidates to increase the search space surrounding high-quality answers. To boost diversity and better explore the solution space, the cloned candidates go through hypermutation, which involves making tiny, random modifications. To make sure that the advantageous mutations are kept, the mutated clones undergo another affinities analysis. The program then uses a three-parent crossover operation to improve the quality of the solution and genetic diversity even further. Segments from each of the three parent solutions—which are chosen based on their affinity—are merged to form new offspring. The recombination procedure improves the overall performance of the algorithm by increasing the inheritance of beneficial qualities and utilizing the strengths of several high-quality solutions. The offspring generated through this crossover inherit characteristics from all three parents, fostering genetic diversity and enhancing the algorithm's ability to find optimal solutions. Through this iterative process of selection, cloning, hypermutation, and three-parent crossover, X3PAIS demonstrates robust optimization capabilities, making it a powerful tool for complex problems such as power loss mitigation in distributed generation networks [19,20].

**Fig. 2:**
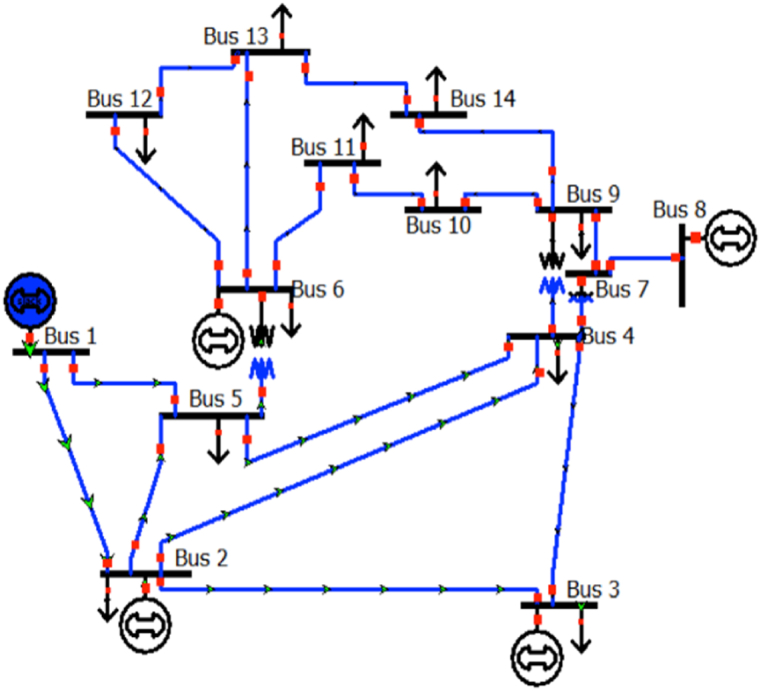
IEEE 14 bus system (Voltage 1000MVA).

This study used simulation programs, MATLAB, and IEEE benchmark system data of a 14-bus system to evaluate the performance of the method (Fig. 2). The benchmark system data used in this study was obtained from IEEE, using a base voltage of 100MVA. The following are the paper's main contributions:

(a) The X3PAIS technique was effectively used in a limited number of applications for the purpose of validation.
  1. Functions of mathematical tests
  2. The process of aeration in a wastewater treatment plant (WWTP)
  3. Design of Planetary Gear Train (PGT)
(b) The X3PAIS algorithm was effectively implemented in an electrical distribution system.

## 2 Background and motivation of this research

### 2.1 Artificial immune system (AIS)

In recent years, there has been a growing interest in the AIS, which is now seeing a significant increase in the area of Artificial Intelligence (AI). Doyne, Perelson, and Varela were the pioneers in developing the first AIS [21]. The primary source of inspiration for AIS is derived from the inherent characteristics of biological immune systems. The immunological system and process exhibit variations based on the specific sorts of invaders. The immune system employs several reaction mechanisms to either neutralize the pathogenic impact or eliminate the invading cell, depending on the specific kind of infection. According to De Castro et al. [22], the concept of AIS draws inspiration from theoretical immunology and the observable functions, principles, and models of the immune system. These principles and models were then applied to the field of engineering problem-solving in 2002. Several typical strategies are used in the utilization of AIS models, including immunological network theory [23], negative selection [24], clonal selection [25], dendritic cells [26], and other others. The Clonal Selection Algorithm (CSA) is the most appropriate approach for optimization objectives when compared to other techniques. While Genetic Algorithms (GA) use crossover and mutation to evolve superior solutions like natural selection. Separately, AIS is strong at maintaining variety, and GA is strong in systematic search. By combining these, the GA-AIS algorithm improves genetic variety and solution quality by utilizing a novel three-parent crossover and AIS's adaptive learning and GA's genetic operations [27,28]. Power loss prevention in distributed generation systems is one of the complex optimization challenges that this hybrid technique successfully addresses.

#### 2.1.1 Clonal selection algorithm (CSA)

The CSA is a stochastic optimization technique that is fundamentally based on the principles of clonal selection theory. Within the field of immunology, the clonal selection hypothesis elucidates the mechanism by which immune cells mount a reaction to a particular antigen that infiltrates the organism. B lymphocytes produce antibodies, which exhibit varying degrees of effectiveness against various antigens. Antibodies with more effectiveness will exhibit a faster rate of proliferation, resulting in the elimination of antigens [25]. De Castro and Von Zuben [29] presented a CSA framework, referred to as CLONALG, in the year 2002. The adaptation of CLONALG included both pattern recognition and optimization challenges. The present program aims to replicate the processes of proliferation, variation, and selection seen in the field of immunology. In order to address optimization difficulties, the CLONALG algorithm considers antibodies as the potential solutions, with a singular antigen serving as the objective function. Meanwhile, affinity is considered the value of the objective function, which is understood as the quality of the candidate solution. In essence, the objective of CLONALG is to identify the optimal antibody for a given antigen by considering its affinity. Below is a description of the clonal selection optimization summary [25].

(a) Produce a randomly selected beginning population of AB, as provided by:

$$P\left(0\right)=\left\{AB_{1}\left(0\right),\ldots .,AB_{n}\left(0\right)\right\}$$

(b) Determine the level of fitness for each AB,

$$P\left(0\right)=\left\{f\left(x_{1}\left(0\right),\ldots .,x_{n}\left(0\right)\right)\right\}$$

(c) All cells within the AB population were cloned. The clone quantity is determined by,

$$N_{c}=\sum_{i=1}^{n}round\left(\frac{\beta .N}{i}\right)$$

where $N_{c}$ represents the total number of clones produced for each antibody, β denotes the multiplicative factor, and N represents the quantity of antibodies.

(d) The clone population should be mutated in order to generate a fully developed clone population with a specified number of children. The mutation rate is determined by $\alpha =e^{-\rho f}$, In this context, α represents the mutation rate, whereas denotes the fitness function value that has been normalized to a value of [0.1]. The newly developed AB is comprised of $P=\{AB_{1},\ldots .,AB_{\delta }\}$.
(e) Assess the affinity values of the population of clones, $P=\{f(x_{1},\ldots .,x_{\delta }\}$. The subsequent iteration of Ab is derived by the use of G=ε+δ selection, wherein the most optimal individuals are chosen from the G population.
(f) Select the most optimal AB to form the new Ab population, $P_{new}=S_{G}P$.
(g) Steps (c-f) are repeated until a pre-defined stopping condition is reached.

Having a uniform nomenclature is crucial when defining CSA, since it is a major aspect of this area of research, as suggested by the inspiration. This language is derived from biological inspiration and pertains to the ideas embodied by algorithms that have been inspired, as seen in Table 1.

**Table 1:** Nomenclature is frequently utilized for clonal selection algorithms.

General	AIS (CSA)
Candidate Solution, Exemplar	Antibody, B-Cell, Lymphocyte
Collection of New Samples, Progeny	Clone
Elitism, Memory	Memory Set, Memory Cell
Generalization	Cross-reactivity
Learning Principle	Clonal Selection Principle
Mutation, Variation	Hyper Mutation
Population, Collection of Samples	Repertoire
Re-sampling Principle, Improvement	Affinity Maturation
Re-sampling, Reproduction	Cloning, Clonal Expansion
Selection	Antigen-Antibody Matching
Solution Quality, Fitness	Affinity, Avidity

### 2.2 Genetic algorithm (GA) with transform of artificial immune system (TRANSAIS)

The AIS method was improved by integrating the notion of cell conjugation, drawing inspiration from the genetic modification seen in bacteria. The aforementioned procedure involves the exchange of genetic material between two bacterial cells that are in close touch. The objective of this study was to optimize the optimal outcome of the AIS algorithm by including the crossover process, hence improving its overall performance. Bacterial transformation may take place either spontaneously or become impacted by artificial factors. This methodology seeks to optimize the overall efficiency of the AIS algorithm. The crossover process involves the exchange of genetic material between two antibodies, resulting in the production of mature antibodies A and B. The objective is to get an antibody that acquires better genes. The Genetic Algorithm (GA) is used in this crossover procedure.

**Fig. 3:**
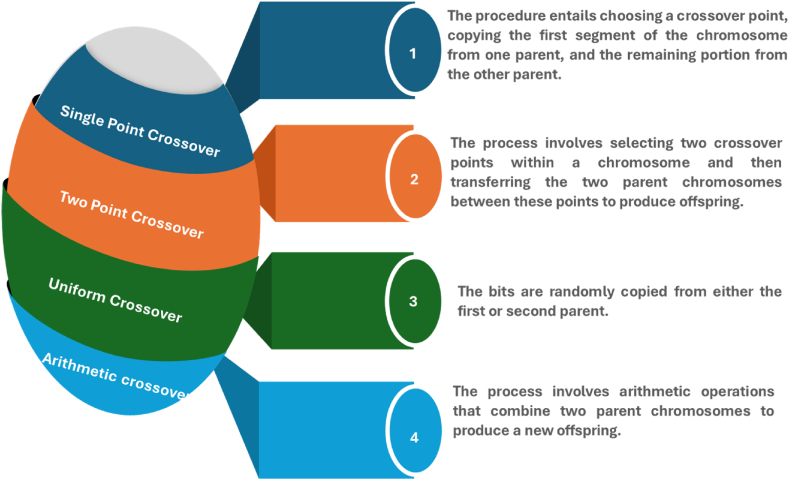
Numerous Crossover techniques.

GA is a branch of the Evolutionary Algorithm (EA) that has shown its effectiveness and success in addressing optimization challenges. It is widely utilized to tackle a wide range of problems. The concept of GA was first introduced by John Holland, a researcher from the University of Michigan, in the early 1960s [30]. GA is a search methodology that conceptualizes a problem and employs a series of operations to traverse the search area. Every chromosome within a population corresponds to a distinct set of genetic material, and a population is composed of these sets. Genetic variations in the population are improved over time via the processes of selection, crossover, and mutations, using the set of procedures used by genetic algorithms [31]. Given the early recognition of GA in EA, a portion of the GA process will be used to improve the AIS process, resulting in the development of TRANSAIS. The crossover procedure from GA will be selected. The purpose of crossover is to provide novel solutions and yield a subset of chosen individuals from the existing population. The process of crossover involves the exchange of genetic material between two parents who possess a single chromosome. There are several techniques for crossover, depicted in Fig. 3.

**Fig. 4:**
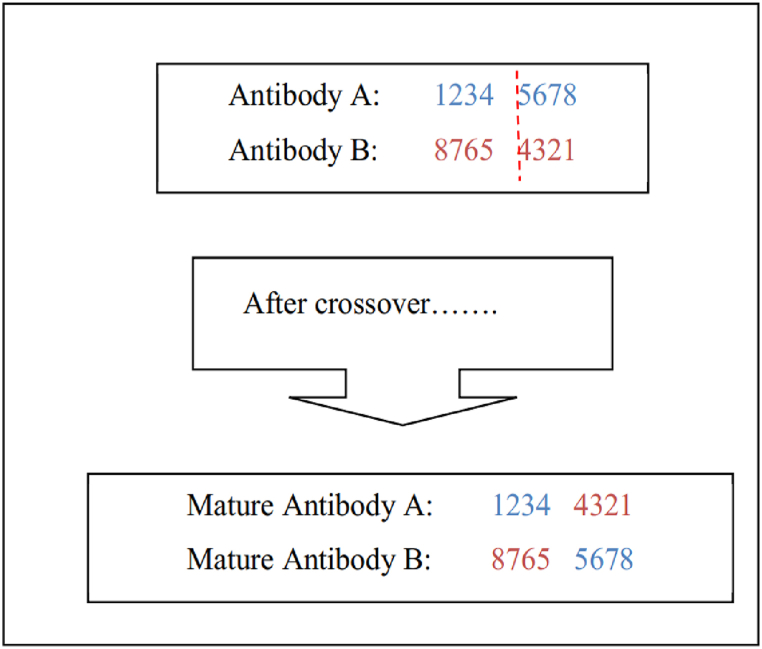
The concept of single-point crossover.

A single-point crossover approach was used in this study to mitigate power loss in an electrical power flow system. The crossing of Antibody A and B yielded two fully developed antibodies. The objective was to develop an enhanced, fully developed antibody that would inherit the advantageous genetic material from the first antibody. The location of the crossing for Antibody A and B is shown by the dashed line in Fig. 4.

**Fig. 5:**
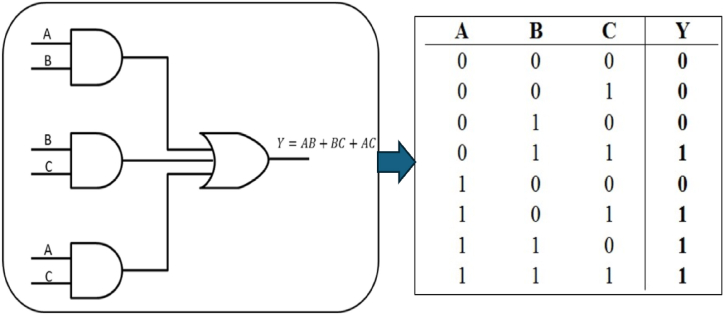
The truth table for the suggested crossover of three parents.

In the process of three-parent crossover, three antibodies are selected as parental entities. The concept is to preserve the superior cell from each parent antibody and combine them to form a new fully developed antibody. During the crossover procedure, a comparison will be made between each bit of the first two parents. In the process of comparison, the bits will be used for generation if they are the same, else the bits of the third parent will be employed. The present study proposes a comparative analysis of the three parents' bits utilizing the AND, and OR logic gates, as seen in Fig. 5.

## 3 Proposed methodology

The proposed optimization technique, based on Artificial Intelligence, aims to optimize power loss in an electrical power flow system by including multiple DGs. The deployment of DGs is evaluated using the IEEE 14-bus system benchmark data. Comprehensive information about power generation units, load profiles, bus voltages, and line impedances is included in the system. The method under consideration underwent testing and validation using field simulation models. AIS and GA have been widely used in solving optimization issues, but a hybrid algorithm is suggested to enhance the current nature-based algorithms. Combining AIS with GA would shorten the time it takes to converge on global minima. The work proposes two (AIS-CSA) based algorithms, X3PAIS and Transform of the Artificial Immune System (TRANSAIS), to enhance the variety and convergence performance of the traditional AIS-CSA algorithm. In order to increase genetic variety, the X3PAIS algorithm was set up with particular parameters, such as population size, cloning rate, mutation rate, and a three-parent crossover strategy. Initializing the test system, creating a population of initial DG configurations, and assessing each potential solution using an objective function that gauges system efficiency and power loss reduction were the steps in the experimental procedure. To make sure the simulation was statistically reliable, several runs were conducted. Every run adhered to a set procedure, which included creating a heterogeneous population, assessing fitness and affinity, choosing superior cloning candidates, utilizing three-parent crossover to produce a new generation of children, and hypermutation to preserve diversity. The performance of each iteration was recorded and analyzed to evaluate the algorithm's effective performance in optimizing the DG system configuration and reducing power losses. The X3PAIS algorithm's practical usability and robustness in real-world power distribution networks are guaranteed by this thorough methodology.

The memory stores 20 high-quality antibodies with a maximum capacity of 100 AB for each repetition. After five repetitions, a cumulative count of 100 AB is stored in the memory. The whole set of Abs stored in memory is compared with the current population to find an additional 20 high-quality AB, referred to as Abext. The procedure continues until a predetermined stopping condition is met.

The following is a step-by-step description of the AIS algorithm:

Step 1 Make a random sample of the population.

Step 2 Determine the fitness level of every population.

Step 3 Make a clone of the whole population.

Step 4 Mutate every population being cloned.

Step 5 Determine the affinity value of every cloned population.

Step 6 Determine the optimal population to comprise the subsequent generation of the random population.

Step 7 Iterate through Step 3, Step 4, Step 5, Step 6 until a stopped condition is satisfied.

**Fig. 6:**
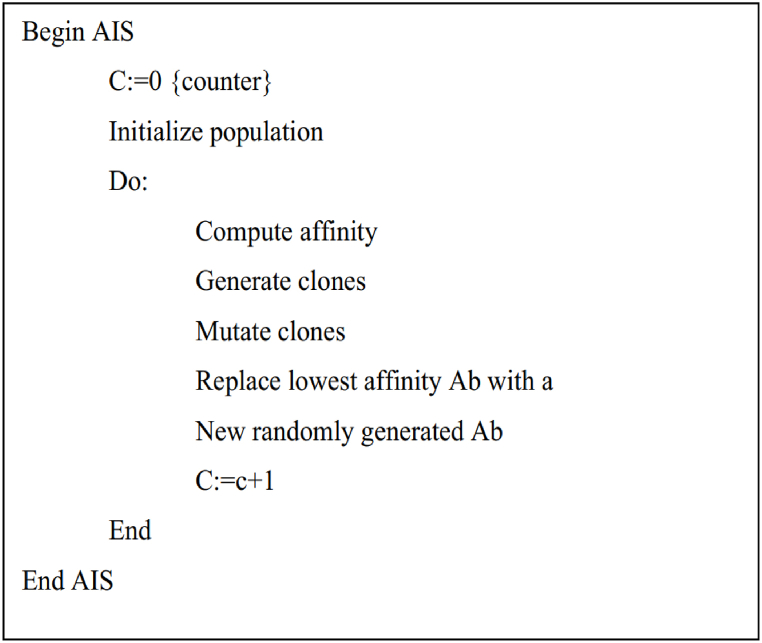
An algorithm for clonal selection (Pseudo-code).

**Fig. 7:**
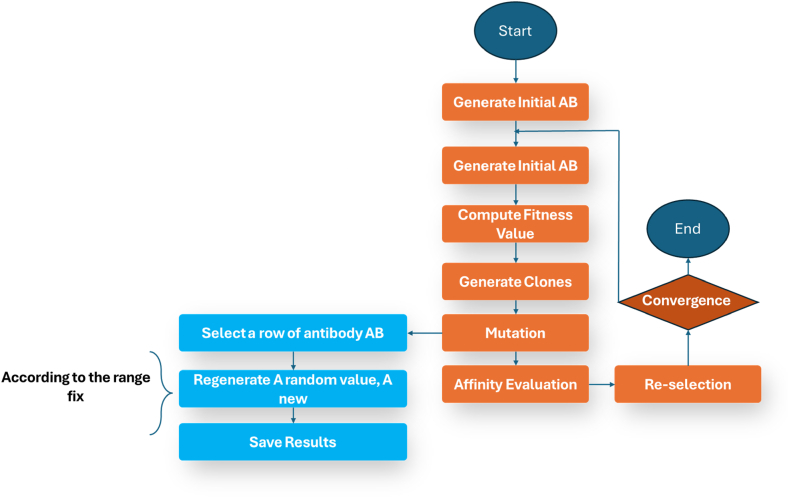
AIS flow chart.

The conventional clonal selection method may be described in pseudocode style, as seen in Fig. 6, and visually represented in a flow chart, as illustrated in Fig. 7.

One of the processes involved in bacterial transformation is conjugation when genetic material is transferred between two bacterial cells that are in intimate touch. The concept of conjugating bacterial cells was used in this study, whereby the crossover procedure was conducted on the more favorable outcomes, hence enhancing the AIS algorithm. However, to guarantee that the two chosen superior genes obtained from the AIS algorithm have the most effective antibodies, the most optimal criterion for the gene population will include cloning and incorporating them into new genes.

**Fig. 8:**
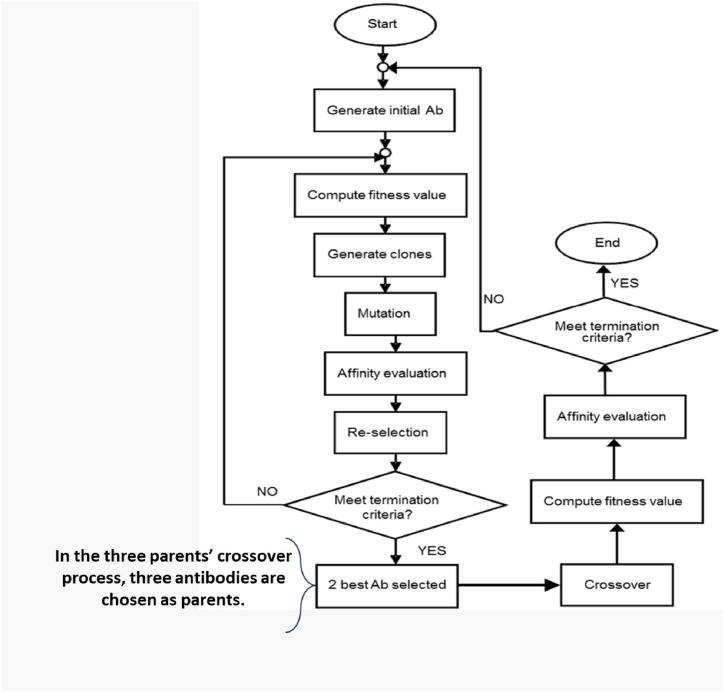
A simplified flowchart illustrating the phases of the TRANSAIS algorithm.

Upon completion of the crossover process, the genetic material of both antibodies was exchanged, resulting in the production of two fully developed antibodies, A and B. Through the process of crossing, a more advanced and refined antibody was acquired, with the anticipation of inheriting superior genes. The process of conjugation will be shown via the use of crossover, which employs the genetic algorithm operator. Fig. 8 provides a simplified depiction of the TRANSAIS process.

### 3.1 Test function

A preliminary validation was conducted on a mathematical function. There are several benchmark functions that possess distinct qualities, which are used for the purpose of comparing and evaluating various types of algorithms. In order to get superior outcomes, several algorithms used the characteristics of the widely used benchmark function. The method is evaluated using eight mathematical test functions in this study. The functions used for testing are Rastrigin function, DeJong function, Griewank function, Ackley function, Axis Parallel Hyper-Ellipsoid function, Move-Axis Parallel Hyper-Ellipsoid function, Rosenbrock function, and Rotate Hyper-Ellipsoid function. These functions are presented in Table 2.

**Table 2:** The eight test functions of the study.

Name	Equation
Rastrigin function	$f_{1}\left(x\right)=\sum_{i=1}^{n}\left(x_{i}^{2}-10\;\cos\left(2\pi x_{i}\right)+10\right)$ Where: −5.12 ≤ $x_{i}$ ≤ 5.12, *i* = 1, …… I, *n*
DeJong function	$f_{2}\left(x\right)=\sum_{i=1}^{n}x_{i}^{2}$ Where: −5.12 ≤ $x_{i}$ ≤ 5.12, *i* = 1, …… I, *n*
Griewank function	$f_{3}\left(x\right)=1+\frac{1}{4000}\;\sum_{i=1}^{n}x_{i}^{2}-\prod_{i=1}^{n}\cos\;\left(\frac{x_{i}}{\sqrt{i}}\right)$ Where: −600 ≤ $x_{i}$ ≤ 600, *i* = 1, …… I, *n*
Ackley function	$f_{4}\left(x\right)=20+e^{-20e}\sqrt[{-0.2}]{\frac{1}{n}}\sum_{i=1}^{n}-e^{\frac{1}{n}\sum_{i=1}^{n}\;\cos\;\left({2\pi x}_{i}\right)}$ Where: −32.768 ≤ $x_{i}$ ≤ 32.768, *i* = 1, …… I, *n*
Axis Parallel Hyper-Ellipsoid Function	$f_{5}\left(x\right)=\sum_{i=1}^{n}{i.x}_{i}^{2}$ Where: −5.12 ≤ $x_{i}$ ≤ 5.12, *i* = 1, …… I, *n*
Moved Axis Parallel Hyper-Ellipsoid Function	$f_{6}\left(x\right)=\sum_{i=1}^{n}{5i.x}_{i}^{2}$ Where: −5.12 ≤ $x_{i}$ ≤ 5.12, *i* = 1, …… I, *n*
Rosenbrock Function	$f_{7}\left(x\right)=\sum_{i=1}^{n}100{\left(x_{i+1}-x_{i}^{2}\right)}^{2}+{\left(x_{i}-1\right)}^{2}=\sum_{i=1}^{n}{5i.x}_{i}^{2}$ Where: −2.048 ≤ $x_{i}$ ≤ 2.048, *i* = 1, …… I, *n*
Rotated Hyper-Ellipsoid Function	$\sum_{i=1}^{n}\sum_{j=1}^{i}x_{j}^{2}$ Where: −65.536 ≤ $x_{i}$ ≤ 65.536, *i* = 1, . . , *n*; *j* = 1, . . , *i*

## 4 Wastewater treatment plant (WWTP) aeration

The WWTP system, which employs aeration to treat wastewater, including effluent, was used to verify the algorithm. To facilitate the biodegradation of contaminants in wastewater, aeration entails the addition of air. Unlike chemical treatment, which employs chemicals to react and stabilize pollutants, this procedure is essential to biological wastewater treatment systems. Aeration is an essential part of WWTP, which treats wastewater from cities and businesses as a secondary step. Through the regulation of Dissolved Oxygen (DO), biochemical oxygen demand, suspended solids concentration, dissolved phosphorus, and suspended phosphorus, it guarantees the safe and effective removal of nutrients. How well microorganisms and nutritional material decompose depends on the oxygen levels in the tanks.

**Fig. 9:**
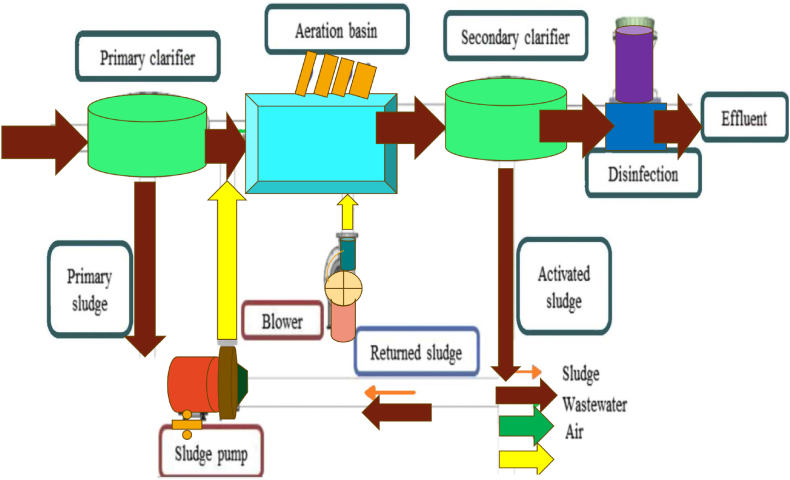
Process flow diagram for wastewater treatment [32].

Wastewater treatment encompasses a wide range of techniques for removing pollutants. Optimal control systems for WWTPs tend to go a little bit beyond the potential for energy savings in industrial settings in order to maintain water quality. Pumps and blowers are examples of energy-intensive equipment used in WWTPs. One possible representation of the WWTP process flow diagram in Fig. 9.

The optimization model below showcases the utilization of DO models and individual effluents formulated from Multi-Adaptive Regression Spline (MARS) [32]. As the plant is being processed, data is collected and the constraint limits for the two models are formulated from there, presented in Table 3.

**Table 3:** Two model equations and the constraint limits.

Model	Equation
Model 1	Z_1_ = min[(w_1_ × y_1_) + (w_2_ × y_2_) + (w_3_ × y_3_) + (w_4_ × y_4_) + (w_5_ × y_5_)] Where: 0 ≤ *y*_1_ ≤ 6.5; 0 ≤ *y*_2_ ≤ 25; 0 ≤ *y*_3_ ≤ 30; 0.2 ≤ *y*_4_ ≤ 1; 0.2 ≤ *y*_5_ ≤ 1; 0 ≤ *w*_1;_*w*_2_, *w*_3_, *w*_4_, *w*_5_ ≤ 1
Mode 2	Z_2_ = min[(w_1_ × y_1_) + (w_2_ × y_2_) + (w_3_ × y_3_) + (w_4_ × y_4_) + (w_5_ × y_5_)] Where: 0 ≤ *y*_1_ ≤ 0.21; 0 ≤ *y*_2_ ≤ 25; 0 ≤ *y*_3_ ≤ 30; 0.2 ≤ *y*_4_ ≤ 1; 0.2 ≤ *y*_5_ ≤ 1; 0 ≤ *w*_1;_*w*_2_, *w*_3_, *w*_4_, *w*_5_ ≤ 1

**Table 4:** Defines the boundaries design variable.

Variable	*t*_*1*_	*m*_*1*_	*b*_*1*_	*c*_*1*_	*r*	*t*_*2*_	*m*_*2*_	*b*_*2*_	*c*_*2*_
**Lower Bound**	13	5	22	4/17	5	13	2	110	4/17
**Upper Bound**	50	9	61	1	10	50	9	140	1

Once the data points have undergone the normalization process, the corresponding constraints and objective function will be altered (see Table 4). In the first model, all weights are set to be equal, whereas in the second model, the weights are redistributed and adjusted for each effluent after normalization.

## 5 Designs for planetary gear train (PGT)

Finally, the method was validated by the implementation of the PGT 2-stage design. PGT was extensively employed in construction machinery and equipment. Presenting strategies for minimizing the volume and minimal weight of PGT for space savings is the major goal of this study.

PGTs provide a number of benefits over conventional parallel axis gear trains, including a lower overall volume, more efficiency, a higher power density, solely torsional responses, and coaxial shafting. They also have numerous kinematic combinations. However, designers have a major challenge when creating PGT systems, and that is the combination of the system's volume and weight. Researchers are therefore concentrating on optimizing the design of PGT in order to reap the benefits mentioned before. The optimization of gear design takes both continuous and discrete factors into account in order to reduce volume. According to conventional wisdom, the best layout considers all nearby discrete points. On the other hand, this might cause the design point to exceed its designated area. Traditional optimization methods often fall into the trap of local minimum search since they are based on gradient algorithms. As a result, it can't compete with more advanced AI methods for producing desirable results. AI is often used to talk about computers that can think and analyze patterns and behavior via the use of computer language [33].

The proposed model used a two-stage PGT gearbox, as presented by T. Chen et al. [34]. The primary goal is to reduce the size of the PGT. Calculated volume for each level of the gear train that includes the sun and planets. The objective is to decrease the volume of the PGT by optimizing the volume for each individual gear. The formulation of the objective function is as follows:

(1) $$V=\frac{\pi }{4}[\left(b_{1}*\left(d_{s_{1}}^{2}+3dp_{1}^{2}\right)\right)+\left(b_{2}*\left(d_{s_{2}}^{2}+3dp_{2}^{2}\right)\right)$$

Where:

$d_{s_{1}}$, $d_{s_{2}}$ = Sun gear circular diameter

$d_{p_{1}}$, $d_{p_{2}}$ = planet gear's circular diameter

The following vector X design variables are developed to optimize the planetary gear train volume using the suggested algorithm:

$$X=\left\{\;x_{1},x_{2}{,x}_{3},x_{4},x_{5},x_{6},x_{7},x_{8},x_{9}\right\}$$

(2) $$=\left\{\;t_{1},m_{1}{,b}_{1},c_{1},r,t_{2},m_{2},b_{2},c_{2}\right\}$$

Where:

$t_{1}$, $t_{2}$*=* Total number of teeth on first- and second-stage sun gear, respectively

$m_{1}m_{2}=$ Modules for the first and second stages, in that order

$b_{1}b_{2}$ = First and second-stage tooth widths, correspondingly

$c_{1}c_{2}$ = Coefficients in the first and second stages, respectively

r = Reduction ratio.

A few restrictions must be established in order to construct the PGT system's parameters and constants. The material model that was selected is 20CrMnMo. The constant and the formulation of restrictions are shown below (Table 5).

**Table 5:** Explanation of the constraint's parameters.

Parameters	Description	1st Stage	2nd Stage
*Z*_*H*_	Node region coefficient	2.22	2.25
*Z*_*E*_	Elastic coefficient	189.98	189.98
*Z*_*e*_	Contact ration coefficient	0.95	0.94
*K*	Load Factor	2.89	2.95
*Y*_*fa*_	Tooth form factor	2.29	2.32
*Y*_*sa*_	Stress concentration coefficient	1.73	1.73
*Y*_*e*_	Contact ratio coefficient	1.12	1.08
*σ*_*c*_	Allowable contact stress	1033.41	1104.47
*σ*_*b*_	Allowable bending stress	499.39	521.53

## 6 Results and discussion

The simulation results are organized as follows: first, the basic AIS and improved based-AIS algorithms solve and optimize a small number of mathematical test functions. Then, the results of the created method are shown to optimize even more test functions.

### 6.1 AIS optimization (mathematical test functions)

**Fig. 10:**
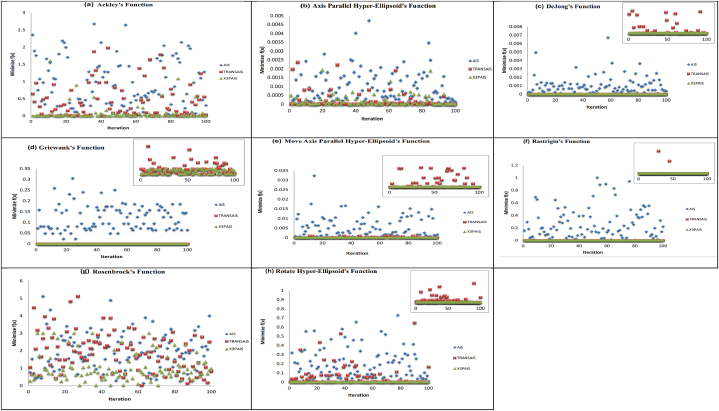
100 Trials Obtained data for eight different functions. (a) Ackley function; (b) Axis Parallel Hyper-ellipsoid function; (c) Dejong function; (d) Griewangk function; (e) Move Axis Parallel Hyper Ellipsoid function; (f) Rastrigin function; (g) Rosenbrock function; (h) Rotate Hyper-Ellipsoid's function.

A total of eight traditional test functions were chosen and replicated in MATLAB to ensure that the AIS, TRANSAIS, and X3PAIS algorithms' functionality. Rastrigin function, DeJong function, Griewank function, Ackley function, Axis Parallel Hyper-Ellipsoid function, Move-Axis Parallel Hyper-Ellipsoid function, Rosenbrock function, and Rotate Hyper-Ellipsoid function. were each of the eight functions listed. The outcomes of 100 trials using the AIS, TRANSAIS, and X3PAIS methods for every test function are shown in Fig. 10. The simulation was run up to 100 iterations since any more iterations would result in a fitness value change to the power of −6, rendering the experiment useless.

From Ackley's function in Fig. 10(a), X3PAIS outperformed all other algorithms, with TRANSAIS and AIS following suit. As opposed to prior findings, the fundamental AIS performs less favorably than the TRANSAIS. In Fig. 10(b), the Axis Parallel Hyper-ellipsoid function demonstrates that the TRANSAIS model attained the highest fitness value, which is similar to that of AIS and X3PAIS. Conversely, the lowest value of X3PAIS is close to that of TRANSAIS. In Fig. 10(c), it can be seen that the minimum value of X3PAIS is equivalent to that of TRANSAIS for Dejong's function. The outcomes of Griewangk's function, as shown in Fig. 10(d), indicate that both TRANSAIS and X3PAIS can achieve the optimal minimum value within a total of 100 iterations. The functions of the Moved Axis Parallel Hyper-ellipsoid in Fig. 10(e) and Rastrigin in Fig. 10(f) exhibit similarities to the Dejong function in Fig. 10(c), where the minimum value of X3PAIS is equivalent to that of TRANSAIS. The function of Rastrigin in Fig. 10(f) exhibits similarities to the function of Dejong in Fig. 10(c) and the function of the Moved Axis Parallel Hyper-ellipsoid in Fig. 10(e), where the minimum value of X3PAIS is identical to that of TRANSAIS. However, while considering Rosenbrock's Function in Fig. 10(g), it is seen that X3PAIS achieves the smallest value compared to the AIS and TRANSAIS algorithms. However, it is important to note that the minimum value is only in the power of −02. The function of the Rotated Hyper-ellipsoid in Fig. 10(h) yields a similar outcome to the function of Ackley in Fig. 10(a). Nevertheless, X3PAIS has marginally superior performance compared to TRANSAIS.

When compared to other algorithms, in comparison to TRANSAIS, the X3PAIS method achieves a lower fitness value, except for the axis parallel hyper-ellipsoid function. The X3PAIS approach yields a nearly zero minimum result for each test function. Table 6 displays the minimum values of f(x), mean, and standard deviation obtained from eight test functions. In terms of population size, number of generations, mutation rate, crossover rate, stopping condition, and clonal rate, all eight test functions are operating under identical conditions for AIS, TRANSAIS, and X3PAIS.

**Table 6:** Test function comparisons for AIS, TRANSAIS, and X3PAIS [35,36] (all values are in 10^−7^).

Function	Minimum [value x E−07]			Mean [value x E−07]			Standard Deviation [value x E−07]		
	AIS	TRANSAIS	X3PAIS	AIS	TRANSAIS	X3PAIS	AIS	TRANSAIS	X3PAIS
**A**	323771	11277	10	9862162	3713335	781290	6810099	4746891	2325998
**B**	43	1	2	10046	1812	1705	8976	4444	3307
**C**	193	0	0	100115	1	0	10522	2	0
**D**	12502	0	0	1174186	1	0	603537	1	0
**E**	73	0	0	52047	1388	0	54512	2821	0
**F**	29035	0	0	2610492	7	0	2376589	54	0
**G**	1393522	132767	122678	19545662	18730811	10218462	10536008	11002873	7823979
**H**	96825	45	4	2026903	540476	2500	1793968	1073312	3658

In this study, the TRANSAIS and X3PAIS algorithms are compared with various algorithms proposed by other researchers, specifically focusing on their minimal value (Table 7). David et al. [37] established many algorithms in the field of machine learning, including the CSA, Particle Swarm Optimization (PSO), the combination of AIS and PSO (PSO-AIS), the Half Best Insertion (HBI) method, and the Single Best Remainder (SBR) algorithm. The performance of PSO-AIS and HBI exhibits a high degree of similarity but with a notable disparity in the pace of convergence shown by PSO-AIS. The performance of CSA may be considered modest with a fitness value of 1.56^−10^. The SBR method, similar to the outcome achieved by CSA, has a favorable fitness value. The fitness value can be efficiently determined by PSO, but it tends to converge too quickly, and the fitness value may be less accurately provided by GA. To address the issue of premature convergence in the optimization of multi-modal functions using the Artificial Bee Colony (ABC) method, Xi Li et al. [38] introduced a hybrid approach known as ABC-BFGS. A unique mutation was used to enable the algorithm to overcome local minima. The test functions used for this study consisted of four well-known multi-valued functions, namely Rosen Brock, Rastrigin, Griewank, and Ackley. The empirical findings indicate that the ABC-BFGS algorithm has a notably rapid convergence rate. The next step, GA, uses the input from the previous generation of AIS, known as hybrid AIS-GA, which was created by M.O. Ali et al. [35]. The objective function's average is calculated for 20 trials. However, it is important to note that the equivalent experiment is conducted for 100 trials and demonstrates the desirable minimum fitness value. As a population-based stochastic optimization method, GA suffers from two main issues: slow convergence speed and a tendency to slip into local optimal spots. For this reason, M.J. Mahmoodabadi et al. [36] address these limitations by presenting an Adaptive Genetic Algorithm (AGA) that incorporates novel mutation and crossover operators. However, compared to the existing research conducted by TRANSAIS and X3PAIS, they demonstrate superior performance in the Griewangk function, Move Axis Parallel Hyper Ellipsoid function, and Rotate Hyper-Ellipsoid function. According to the eight benchmark functions, X3PAIS outperforms the regular AIS in terms of achieving the best possible minimum value. Previous findings demonstrate that X3PAIS outperforms other researchers' algorithms in comparison to the eight test functions' worth of simulations. David et al. [39] evaluated the hybrid PSO-AIS algorithm and a novel external memory CSA-based scheme termed EMCSA algorithm on several test functions to validate the current techniques (see Table 8).

**Table 7:** Examining other researchers' minimum f(x) values for test functions [[37], [38], [35], [36]] (all values are in 10^−7^).

Minimum f(x) [value x E−07]	Function							
	A	B	C	D	E	F	G	H
**X3PAIS**	9.536773	1.61191	0.0000014	0	0.00000853	0.0022553	122678.4	4.023314
**TRANSAIS**	11277.33	1.029968	0.0000014	0	0.00000853	0.0022553	132767.1	44.70348
**HBI [47]**	–	–	12.3	0.0966	–	7.57	–	–
**SBR [47]**	–	–	0.00000008	0.00000287	–	0.0000186	–	–
**CSA [47]**	–	–	0.0000396	0.0000197	–	0.00156	–	–
**PSO [47]**	–	–	0.00000057	0.00000004	–	0.00000876	–	–
**PSO-AIS [47]**	–	–	95.6	1050	–	28.9	–	–
**GA [47]**	–	–	2.64	1.36	–	111	–	–
**AIS [48]**	3572050	–	–	14400	1540000	5880000	3078000	25400
**GA [48]**	139000	–	–	220	2940	938000	563000	353
**Hybrid AIS-GA [48]**	25500	–	–	40.2	54.2	1900	171000	68.9
**ABC [49]**	54072000	–	–	–	–	0	144000	–
**BFGS [49]**	195449000	–	–	–	–	2298339000	7078243000	–
**ABC-BFGS [49]**	0	–	–	–	–	0	0	–
**AGA [50]**	0.00000004	–	–	0	–	0	264830000	–

A: Ackley's function; B: Axis Parallel Hyper-ellipsoid function; C: Dejong function; D: Griewangk function; E: Move Axis Parallel Hyper Ellipsoid function; F: Rastrigin function; G: Rosenbrock function; H: Rotate Hyper-Ellipsoid's function.

**Table 8:** Comparing minimal volume, V, with other researchers.

	Minimum (V) [value x E+05]
**X3PAIS**	22.1317
**TRANSAIS**	22.1306
**AIS**	22.1320
**GA** [33]	120.4459
**PSO** [29]	22.2450
**DE** [32]	145.5583
**RCGA [52]**	3.8449

**Table 9:** Power loss results with and without DG installation.

DG Case	Location	Capacity (MW)	Power Loss (MW)	Percentage Reduction (%)
Without DG	–	–	10.5900	–
2 DGs	3	0.9288	1.2984	87.7389
	8	0.7958		
3 DGs	8	0.8254	1.2910	
	1	0.5214		
	3	0.9507		
4 DGs	8	0.2652	1.0789	89.8118
	3	0.7015		
	6	0.1732		
	2	0.0108		

DG Case	Location	Capacity (MW)	Power Loss (MW)	Percentage Reduction (%)
*without DG*	–	–	10.5900	–
*2 DGs*	3	0.9288	1.2984	87.7389
	8	0.7958		
*3 DGs*	8	0.8254	1.2910	87.8094
	1	0.5214		
	3	0.9507		
*4 DGs*	8	0.2652	1.0789	89.8118
	3	0.7015		
	6	0.1732		
	2	0.0108		

### 6.2 WWTP aeration process

**Fig. 11:**
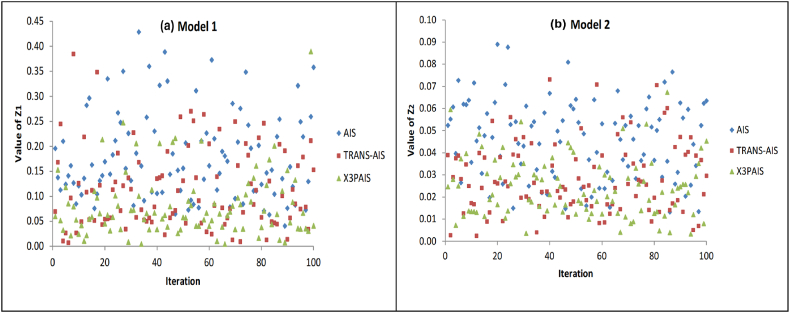
100 Trials Obtained data for 2 models.

The suggested technique will be used in two optimization models in order to minimize the effluents of the aeration process. The simulation in this article uses the MATLAB programming language. Both models are being tested under identical conditions, including generation number, population size, cloning rate, mutation rate, crossover rate, and halting condition for all algorithms. Fig. 11 displays the outcomes of 100 simulations for the two models, using the AIS, TRANSAIS, and X3PAIS algorithms for optimization. According to the data, the X3PAIS method has a minimum value that is closer to 0 compared to other algorithms.

**Fig. 12:**
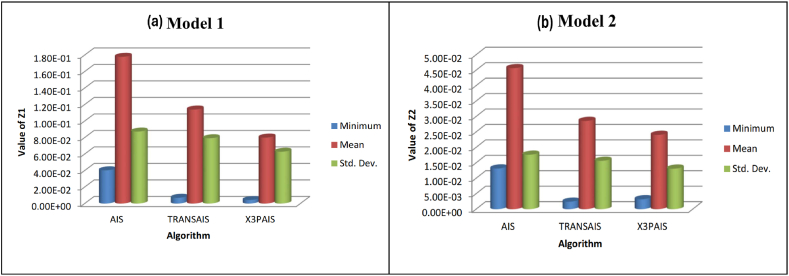
Minimum, Mean, and Standard Deviation Z1 value comparisons utilizing AIS, TRANSAIS, and X3PAIS.

Fig. 12 shows the results of minimizing the Z-value, mean, and standard deviation for two different models. This simulation demonstrates that X3PAIS achieved a better outcome than other algorithms when tested with it. The Water Quality Indicators (WQIs) determined by this study include DO, TSS, TSP, and TDP.

In order to examine how well the suggested AI algorithm reduces WWTP effluents, this simulation uses two different models. When compared to the industry-standard AIS algorithm, the findings demonstrate that the suggested method is superior and gets closer to the optimal value. Comparing X3PAIS algorithm performance to that of regular AIS and TRANSAIS, it is better, but not exceptional. To make X3PAIS even better, need to reevaluate its settings and selection criteria.

Future studies will optimize the energy usage of WWTP systems using the findings of this simulation. For an ideal water quality situation, it influences water quality and vice versa.

### 6.3 Two-stages PGT design optimization

**Fig. 13:**
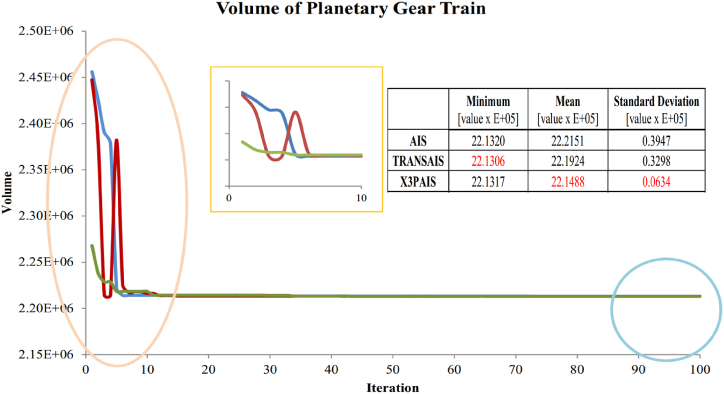
The value of the objective function (V) is determined by each algorithm [20].

Optimizing a multi-stage planetary gear train is a challenging task due to the inclusion of design factors that may be classified as integer or real. The objective of this simulation is to reduce the volume of PGT and minimize its weight in order to save space using a devised algorithm. The minimal volume of the two-stage PGT was determined and optimized via the design of their respective bounds. Fig. 13 displays the comparison of minimum, mean, and standard deviation among AIS, TRANSAIS, and X3PAIS. The findings indicate that X3PAIS achieves superior outcomes compared to regular AIS and TRANSAIS.

A comparison was made between the minimum values obtained in the simulation and those reported by other studies, as shown in Table 5. The use of GA was employed by A. Sri Harsha et al. [34], Kaoutar Daoudi et al. [40] employed PSO, Differential Evolution (DE) was utilized by TianPei Chen et al. [41], and the Real Coded Genetic Algorithm (RCGA) was presented by Paridhi Rai et al. [42]. The algorithm created in this study, X3PAIS, is similar to existing algorithms.

The simulation above clearly illustrated and demonstrated the similar, if not greater, performance of the created method. The method was implemented in the PGT design to minimize the volume. The X3PAIS algorithm demonstrates superior performance in achieving the optimal minimum value in the design of PGT, surpassing the findings of previous studies.

### 6.4 Power loss minimization

The study utilized the X3PAIS algorithm to allocate multiple DG units to minimize power loss in the distribution system. A total of 100 simulations were conducted using MATLAB software. The analytical approach evaluates the optimal size and location of numerous DG units by systematically deploying DG units of varied sizes (ranging from minimum to maximum) at each bus. The losses are then estimated for each scenario.

**Fig. 14:**
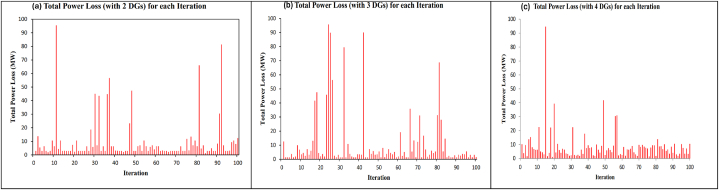
100 testing results after installing 2–4 DG units for complete power loss. (a) Total power loss with 2DG unit. (b) Total power loss with 3DG unit. (c) Total power loss with 4DG unit.

**Fig. 15:**
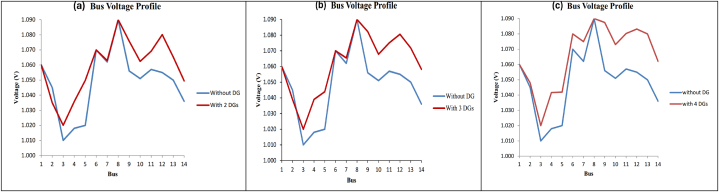
Bus voltage profile for IEEE14 bus test system with 2–4 DG units. (a) (2-DG installation) profile at 84th iteration. (b) (3-DG installation) profile at 96th iteration. (c) (4-DG installation) profile at 19th iteration.

Based on the analytical technique, it has been determined that the optimal placement for the two units of DG is at bus 3 and bus 8. The size of the DG at bus 3 is 0.93 MW, while the size of the DG at bus 8 is 0.80 MW. The losses in the system for this scenario are minimal and the overall power losses is decreased by 87.7 %. The findings of total power losses of the system after the installation of 2–4 units of DG are shown in Fig. 14 (a). Fig. 15(a) shows the bus voltage (installation of 2 DG units) profile at the 84th iteration with the lowest total power losses (1.2984 MW). The optimal placement for the 3 units of DG is on bus 8, bus 1, and bus 3. The size of DG on bus 8 is 0.83 MW, on bus 1 is 0.52 MW, and on bus 3 is 0.95 MW. The losses in the system for this scenario are minimal, resulting in a reduction of overall power losses by 87.8 %, shown in Fig. 14(b). Fig. 15(b) shows the bus voltage (installation of 4 DG units) profile at the 96th iteration with the lowest total power losses (1.2910 MW). The optimal placement for the four units of DG is on buses 8, 3, 6, and 2. The relative sizes of the DG units are 0.27 MW, 0.70 MW, 0.17 MW, and 0.01 MW. The system experiences minimal losses in this situation, resulting in a reduction of total power losses by 89.8 % (Fig. 14 (c)). Fig. 15(c) shows the bus voltage (installation of 5 DG units) profile at the 19th iteration with the lowest total power losses (1.0789 MW).

Table 5 shows that the suggested approach has decreased the overall power loss of the IEEE 14 bus distribution system by installing 2–4 units of DG. The table presents the data indicating that the installation of 4 DG units at bus 2, 3, 6, and 8, with corresponding loads of 0.0108 MW, 0.7015 MW, 0.1732 MW, and 0.2652 MW, successfully reduces about 89 % of the total power losses in the distribution system. While the suggested approaches provide an ideal location for DG, they also result in the lowest power losses when the DG is of optimal size. The findings demonstrate the reliability of the suggested algorithm approach.

### 6.5 Discussion

This section presents the validation findings of the algorithm that was designed. The validation application consists of eight mathematical test functions, namely Rastrigin, Dejong, Griewank, Ackley, Axis Parallel Hyper-Ellipsoid, Moved Axis Parallel Hyper-Ellipsoid, Rosenbrock, and Rotated Hyper-Ellipsoid functions. These functions are used to assess the aeration process in WWTP and PGT design. The shown validation findings are similar, but not superior, across all sectors. Once the validation process is over, the X3PAIS algorithm is used in the primary application, which is an electrical power flow system. Its purpose is to determine the most efficient position and size within the distribution system in order to minimize power losses. The optimal placement of several DG units is chosen to minimize power losses and improve the voltage profile of the system. The ideal size of DG results in a substantial decrease in loss and a suitable enhancement in the voltage profile. A test was conducted on the method for the IEEE 14 bus system, and the obtained results were deemed acceptable. Optimizing the placement and size of DGs at different sites enhances the security, reliability, and efficiency of the power system. The implementation of four units of DGs in the IEEE 14-bus distribution system resulted in a noticeable reduction in power losses. The three-parent crossover strategy, which increases genetic variety and solution quality, is a unique blend of AIS and GA principles that makes the X3PAIS algorithm perform better than other algorithms. With the help of this hybrid strategy, X3PAIS is better able to avoid local optima and conduct a more complete exploration of the solution space. Particular elements affecting these outcomes include GA's strong search and optimization capabilities, which quicken convergence to ideal solutions, and AIS's adaptive learning capacity, which preserves a wide pool of superior answers. Furthermore, by combining advantageous features from several systems, the innovative crossover mechanism improves system efficiency and power loss reduction. Hence, the ×3PAIS strategy is a superior solution compared to other ways for addressing the DG placement and size issue, with the objective of minimizing system losses and improving the voltage profile. For instance, the X3PAIS algorithm achieved over 89 % power loss reduction, a significant improvement over the typical 70–80 % reduction seen in previous studies using conventional methods.

However, the increased complexity of X3PAIS can lead to some limitations like longer computation times, which could be considered a weakness in time-sensitive applications. Despite this, the substantial gains in solution quality and robustness make X3PAIS a valuable advancement in the optimization of DG systems, highlighting its superiority over traditional approaches. Future research could explore the application of the X3PAIS algorithm to various power distribution network configurations, including different topologies and load profiles, to assess its versatility and robustness across diverse scenarios. Additionally, integrating real-time data and adaptive parameters could enhance the algorithm's performance in dynamic environments, making it more applicable to smart grid technologies. Another promising direction is the incorporation of renewable energy sources, such as solar and wind power, into the optimization framework. This would include creating models that take into consideration these energy sources' erratic and unpredictable characteristics, therefore improving the algorithm's suitability for use in contemporary power networks. To further enhance the quality of solutions and speed of convergence, researchers can further look into hybrid approaches that combine X3PAIS with other optimization techniques like Differential Evolution (DE) or Particle Swarm Optimization (PSO). It would also be possible to facilitate the industry's adoption of the GA-AIS technique by performing substantial real-world case studies and pilot projects, which would offer insightful information on the system's operations and practical use.

## 7 Conclusion

In the pre-evolutionary era, a variety of approaches existed for addressing optimization issues. Several prominent methods include the enhanced LLagrange multiplier approach, Powell's zeroth order method, Fletcher and Reeves Conjugate Gradient method, and the Branch and Bound algorithm. Nevertheless, the use of nature-based algorithms has gained prominence as a result of the limitations of algorithms, particularly in addressing intricate optimization challenges with regard to speed and accuracy. Evolutionary algorithms, such as the Genetic algorithm and Clonal Selection algorithm, have gained popularity in addressing practical real-world issues because of their simplicity and resilience.

Each evolutionary method has limitations that are contingent upon the specific sort of optimization task at hand. To address the challenges encountered, the present study used a straightforward approach referred to as hybridization. Essentially, the advantageous characteristics of AIS and GA were merged to create an improved hybrid model (X3PAIS). The simulation findings demonstrate that, when applied to a mathematical test function with a single dimension, the Hybridization of two algorithms exhibits superior performance compared to the basic AIS. Regrettably, the performance of TRANSAIS deteriorates for multidimensional test functions. In this research, an enhanced AIS is presented to optimize the efficiency of current evolutionary algorithms used for solving intricate mathematical functions with many dimensions and modes. The AIS approach was also devised to mitigate power losses in the power distribution flow system by the use of multiple dispersed generators. According to the simulation findings, X3PAIS demonstrates superiority over alternative algorithms across the majority of test functions.

Nevertheless, the algorithm now under development may potentially be improved for future endeavors. Several novel optimization techniques have been created, potentially enhancing the precision and efficiency of this study.

AIS is now being investigated for its potential to address intricate mathematical challenges, as well as practical applications in several domains, including signal processing, picture compression, data security, virus scanners, and numerous others. Therefore, it is crucial to enhance the algorithm's performance to align with the future objectives of this project.

## CRediT authorship contribution statement

**Oday A. Ahmed:** Writing – original draft, Visualization, Software, Methodology, Funding acquisition, Data curation. **K.H. Chong:** Writing – original draft, Visualization, Supervision, Software, Resources, Project administration, Formal analysis, Conceptualization. **S.P. Koh:** Writing – review & editing, Writing – original draft, Visualization, Supervision, Resources, Methodology, Investigation, Funding acquisition, Conceptualization. **Chong Tak Yaw:** Writing – review & editing, Writing – original draft, Validation, Resources, Methodology, Investigation, Formal analysis. **Jagadeesh Pasupuleti:** Writing – review & editing, Visualization, Resources, Formal analysis, Conceptualization.

## Declaration of competing interest

The authors declare that they have no known competing financial interests or personal relationships that could have appeared to influence the work reported in this paper.

## Figures and Tables

**Table 1:** Examining other researchers' mean f(x) values for test functions [36,39].

Mean f(x) [value x E−07]	Function							
	A	B	C	D	E	F	G	H
**X3PAIS**	78128.9697	170.53	0.000006	0.04119	0.000044	0.000442	1021846.18	249.961
**TRANSAIS**	371333.462	181.171	0.066706	0.08373	138.781835	0.742131	1873081.1	54047.6
**CSA [51]**	122830	0.59	0.61	0.2	110	2109170	4487520	18
**EMCSA [51]**	1629300	15.6	9.5	12.4	2300	5586520	4007330	2900
**PSO [51]**	1330070	126530	115650	19400	1996600	12105700	11414000	1722300
**PSO-AIS [51]**	1473660	0.69	0.82	0.26	170	4888300	2291040	30
**GA [51]**	17310	150	190	22	2390	2118600	6056020	2930
**AGA [50]**	0	*	*	0	*	0.003978	27034000	*

**Table 2:** Examining other researchers' Standard Deviation f(x) values for test functions [36,39].

Standard Deviation f(x) [value x E−07]	Function							
	A	B	C	D	E	F	G	H
**X3PAIS**	2325998.267	3306.905231	0.0000866	0.3861412	0.00073988	0.00883725	7823979.12	3657.853521
**TRANSAIS**	4746890.88	4444.085982	10521.80791	0.91246675	2820.845036	53.92661316	11002872.67	1073311.606
**CSA [51]**	3629900	3.9	4.8	1.3	1100	15161700	13898400	130
**EMCSA [51]**	7043900	203	74	117	7500	15683000	21816200	16000
**PSO [51]**	8346000	811000	770900	226300	10889500	56180200	37569300	24683100
**PSO-AIS [51]**	8552500	4.6	6.3	2.1	3300	10940400	13040000	330
**GA [51]**	30400	710	1400	160	7500	11876700	21891300	16500
**AGA [50]**	0.00000002	*	*	0	*	0.2178	2930000	*
